# A Hybrid Readout Solution for GaN-Based Detectors Using CMOS Technology †

**DOI:** 10.3390/s18020449

**Published:** 2018-02-03

**Authors:** Preethi Padmanabhan, Bruce Hancock, Shouleh Nikzad, L. Douglas Bell, Kees Kroep, Edoardo Charbon

**Affiliations:** 1 AQUA Laboratory, École Polytechnique Fédérale de Lausanne (EPFL), 2000 Neuchâtel, Switzerland; edoardo.charbon@epfl.ch; 2 Jet Propulsion Laboratory, California Institute of Technology, Pasadena, CA 91109, USA; bruce.hancock@jpl.nasa.gov (B.H.); shouleh.nikzad@jpl.nasa.gov (S.N.); lloyddoug.bell@jpl.nasa.gov (L.D.B.); 3 AQUA Laboratory, Delft University of Technology, 2628 CD Delft, The Netherlands; H.J.C.Kroep@student.tudelft.nl

**Keywords:** Gallium nitride (GaN), avalanche photodiodes (APDs), UV imaging, CMOS technology, capacitive transimepdance amplifier (CTIA), hybridization, 3D integration

## Abstract

Gallium nitride (GaN) and its alloys are becoming preferred materials for ultraviolet (UV) detectors due to their wide bandgap and tailorable out-of-band cutoff from 3.4 eV to 6.2 eV. GaN based avalanche photodiodes (APDs) are particularly suitable for their high photon sensitivity and quantum efficiency in the UV region and for their inherent insensitivity to visible wavelengths. Challenges exist however for practical utilization. With growing interests in such photodetectors, hybrid readout solutions are becoming prevalent with CMOS technology being adopted for its maturity, scalability, and reliability. In this paper, we describe our approach to combine GaN APDs with a CMOS readout circuit, comprising of a linear array of 1 × 8 capacitive transimpedance amplifiers (CTIAs), implemented in a 0.35 µm high voltage CMOS technology. Further, we present a simple, yet sustainable circuit technique to allow operation of APDs under high reverse biases, up to ≈80 V with verified measurement results. The readout offers a conversion gain of 0.43 µV/e^−^, obtaining avalanche gains up to 10^3^. Several parameters of the CTIA are discussed followed by a perspective on possible hybridization, exploiting the advantages of a 3D-stacked technology.

## 1. Introduction

Ultraviolet (UV) wavelengths have been of particular interest in space applications for their importance in the study of planetary bodies and their atmospheres [1,2]. The UV spectrum provides essential information of various elements and compounds, helping us better understand the nature and habitability of the planetary bodies [3]. All of these are not possible without high-performance photodetectors, with photon counting capability necessary for faint object detection. Traditional instruments incorporate microchannel plates and photomultplier tubes, with high sensitivity in the UV region. Silicon-based detectors have also seen growing interest for their easier scalability for mass production and reliability, however, they are mainly limited by their achievable signal-to-noise ratio (SNR) in UV wavelengths [4].

Addressing the aforementioned challenges, recently, Jet Propulsion Laboratory (JPL) in California developed avalanche photodiodes (APDs) based on Gallium nitride (GaN) for imaging applications in the UV region, thanks to the tailorable bandgap possible in III-Nitride materials [4]. GaN, with its bandgap of 3.4 eV provides inherent out-of-band rejection in the visible wavelengths, thus achieving solar-blind UV sensitivity. Reverse-biased typically at high voltages (≈80 V), the GaN APDs generate currents on the order of hundreds of picoamperes in proportional-mode, while avalanching to more than a few microamperes beyond the breakdown voltage.

Most often, high-performing detectors such as these are not ready for immediate utilization, mainly because of the lack of integration to an imaging system. This limitation, thus, imposes the fundamental need to have an appropriate readout solution [5]. The growing role of such detectors lead to more and more solutions around a hybrid sensor design, facilitating applications which span over UV to infrared (IR) wavelengths. Complementary metal-oxide-semiconductor (CMOS) technology is being considered as a readout solution for such photodetectors, thanks to its scalability, easy integration, and reliability [6,7]. Evolving from 2D integration, hybridization is gathering more attention with the advent of 3D-integrated technology [8,9,10,11].

We have been collaborating with JPL to develop a suitable readout for the III-Nitride based detectors. In [4], the device and process-level description of these detectors have been covered in depth, while, in this work, we present results of the readout circuit implemented for these detectors extended from [12]. We describe our proposed approach to combine CMOS readout circuit with GaN APDs, operated in the proportional mode. We discuss the implemented circuit-level concepts for APDs, particularly useful to sustain high avalanche voltages without damaging the low-voltage CMOS circuit. A perspective towards possible readout hybridization is also discussed, showing a promising future for a number of imaging applications.

## 2. Gallium Nitride APDs

**Figure 1:**
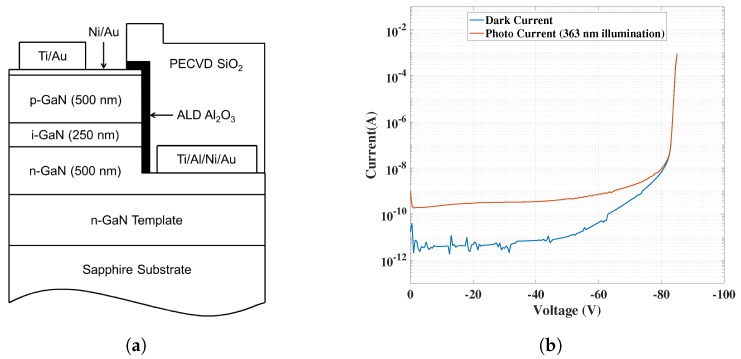
GaN APDs developed at JPL: (**a**) device geometry of a GaN APD used [4]; and (**b**) typical I–V characteristics (redrawn from [4]).

GaN APDs designed at JPL have been used to achieve solar-blind UV sensitivity with high gain and responsivity. At 360 nm, they have an external quantum efficiency of about 60% and four orders of magnitude out-of-band rejection ratio. The avalanche gain in these devices reaches 10^5^, competing with state-of-the-art GaN devices [4]. In the proportional-mode of operation, these APDs generate equivalent currents on the order of 100–200 pA with a reverse bias of ~70 V, as seen in Figure 1b. Beyond 70 V, which is typically their breakdown voltage, the diodes enter into the Geiger mode of operation. In Figure 1a, one can also see the device geometry of a GaN APD—a p-i-n structure also used for testing with the readout circuit implemented in this work.

## 3. Design of CMOS Readout Integrated Circuit

The previous section briefly covered the details of the GaN APDs along with their I–V characteristics in the proportional mode of operation. Utilization of GaN detectors for various UV applications imposes the very fundamental need to have an appropriate readout solution with amplifying capabilities and high SNR. The sensor proposed here is based on a hybrid structure with the detectors processed in III-Nitride material and the readout circuit implemented in CMOS technology. The two most important requirements addressed in this work are the readout’s ability to handle high avalanche voltages (up to 80 V) and quenching of any possible breakdown in the APDs.

**Figure 2:**
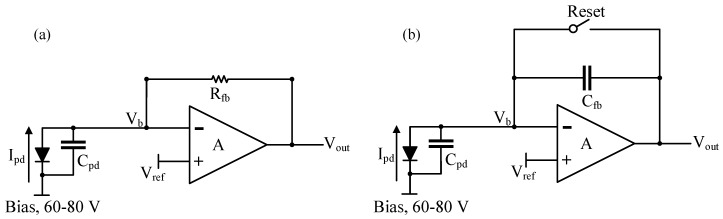
(**a**) Resistive transimpedance amplifier (RTIA); and (**b**) capacitive transimpedance amplifier (CTIA).

Transimpedance amplifier (TIA) circuit became a straightforward choice in order to amplify the low-amplitude photodiode currents, I_pd_ (in this case, on the order of hundred picoamperes). A TIA with a resistive feedback (shown in Figure 2a) will generate an output voltage directly proportional to the feedback resistor according to the equation: V_out_ = I_pd_ × R_fb_. The advantage of choosing the gain by simply adjusting R_fb_ is however, limited by the area occupied by the resistor itself which in turn also limits the achievable transimpedance for a given silicon area. Thus, a TIA with capacitive feedback (shown in Figure 2b) was chosen instead. A CTIA-based topology provides gain which is inversely proportional to C_fb_, which also reduces the area constraints when compared to a RTIA.

### 3.1. Capacitive Transimpedance Amplifier (CTIA)

**Figure 3:**
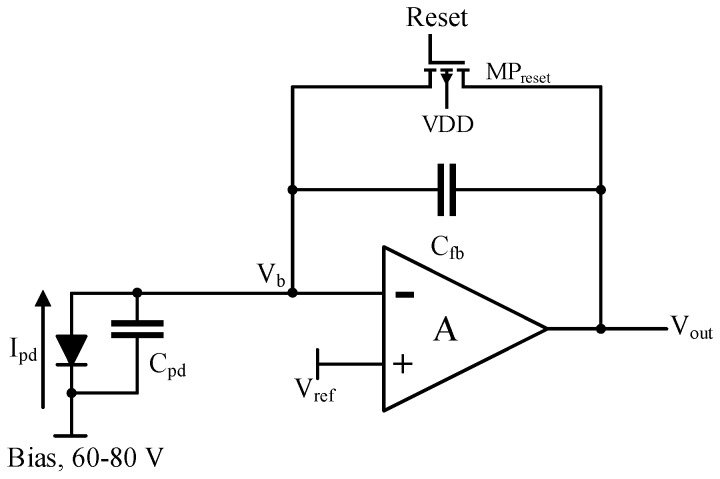
CTIA block diagram.

A photodiode circuit model was derived from the GaN APD characteristics, comprising of APD capacitance approximately 1–3 pF and equivalent APD currents on the order of 100–200 pA under a reverse bias of about 80 V. A conventional CTIA with a parallel reset using a PMOS transistor was designed, as shown in Figure 3.

The CTIA integrates the incoming photodiode current (I_pd_) on the feedback capacitor (C_fb_) in order to generate an equivalent observable voltage at the output. The low-frequency gain is set by the ratio of photodiode capacitance (C_pd_) and the feedback capacitance (C_fb_). In the frequency domain, this behavior can be represented as,

(1) $$I_{pd}\left(s\right)+\frac{V_{out}\left(s\right)}{A}\left(sC_{fb}+sC_{pd}\right)=V_{out}\left(s\right)sC_{fb}$$

where A is open loop gain of the CTIA. In the time domain, this approximates to the following equation, when A >>$\frac{C_{pd}}{C_{fb}}$,

(2) $$V_{out}=\frac{1}{C_{fb}}\int I_{pd}dt$$

The readout operation begins with resetting of the feedback capacitor C_fb_ by switching the voltage at the gate of the reset transistor MP_reset_ before every integration. Immediately after the release of the reset switch (as Reset → VDD), the photodiode current starts to flow in and integrates on the feedback capacitor and the output of the amplifier starts to drop from the initial value V_ref_ set by the reset transistor (when Reset = 0) which defines the DC operating points of the CTIA. The negative feedback of the amplifier maintains the input node at virtual ground under infinite gain. However, due to the finite gain (A) of the CTIA, there is also a small rise in the voltage level at the input node V_b_.

#### 3.1.1. Design Challenge- High Voltage Problem and Quenching

One of the major challenges while designing a readout for the GaN devices is the requirement to accommodate high reverse bias voltages applied on these devices. This implies providing a means to isolate the low-voltage CMOS circuitry (operating, typically up to 3.3 V) from the high bias voltages. Since the GaN devices were fabricated in a number of flavors at the process level, there was also a possibility of variation in the breakdown voltage of these devices. Another challenge is hence, posed by any potential avalanche breakdown which could occur under such a bias due to the presence of high electric fields in the device. In such conditions, carrier multiplication must also be quenched to avoid damage to the APD and also to permit next photon detection. Additionally, the large current flow during avalanche separates charges, creating a dipole and collapsing the voltage across the APD. This leaves the high voltage directly across the readout.

**Figure 4:**
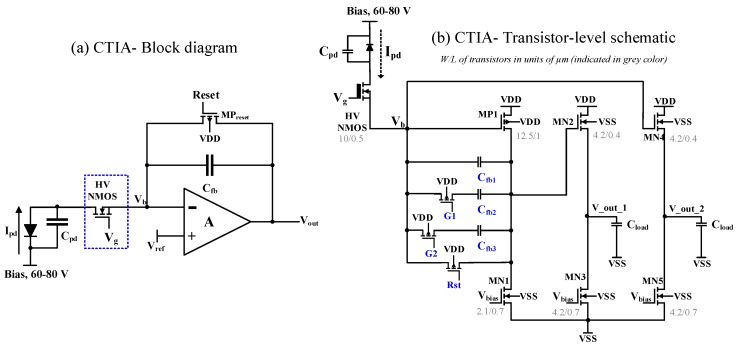
(**a**) CTIA block diagram with HV NMOS transistor; and (**b**) CTIA transistor-level schematic.

Both of the above challenges were addressed by providing a protection circuit using a high voltage (HV) NMOS transistor at the input of the CTIA (outlined in blue colour in Figure 4a).

Initially, the gate voltage of the HV NMOS is set such that it biases the transistor to operate in its ohmic region. In this region, there is very little voltage drop across the HV NMOS; thus, the detector receives most of the applied bias voltage. When the CTIA saturates after completing the integration process, the input voltage, V_b_, starts rising as the incoming photodiode current can no longer integrate on the feedback capacitance. Especially, during an avalanche breakdown when the CTIA saturation will occur rapidly, the rising input voltage can reach damaging levels if it is neglected.

Introducing the HV NMOS in the input path reduces the gate-to-source voltage V_gs_ of the HV NMOS over rising levels of the node V_b_. The HV NMOS thus shuts off when its V_gs_ becomes lower than its threshold voltage, thus isolating the low-voltage CMOS readout circuit from the APD stage. Following this, the photodiode current will end up discharging the photodiode capacitance C_pd_ and no longer flow into the CTIA, eventually also reducing the bias across the APD, which is specifically useful in the quenching mechanism whenever there is avalanche current surge.

The transistor-level schematic of the CTIA is shown in Figure 4b. The core of the CTIA is a common-source amplifier (transistors MP1 and MN1). Given that the photodiode current is generated out of a p-on-n type APD device, a PMOS input (MP1) is used as the gain transistor of the common-source stage. This stage is followed by a NMOS source follower (transistors MN2 and MN3) which acts as an output buffer to avoid any voltage degradation at the output, capable of driving low impedance loads. There is another source follower stage (transistors MN4 and MN5) connected to the input node V_b_ to allow observation of rising voltage levels. The readout provides variable gain by featuring four effective feedback capacitances and two switches, G1 and G2 in Figure 4b, obtained such that C1 = C_fb1_, C2 = C_fb1_ + C_fb2_, C3 = C_fb1_ + C_fb3_, C4 = C_fb1_ + C_fb2_ + C_fb3_. The relative design values were selected such that C2 − C1 = 100 fF, C3 − C2 = 200 fF, C4 − C3 = 100 fF. The bias voltages V_bias_ for the load transistors is generated using a simple current mirror. The PMOS and NMOS transistors in this technology were characterized by conducting device simulations on Cadence software. Transistor-level parameters such as the intrinsic gain of the transistor g_m_/g_ds_, the transconductance-to-drain current ratio g_m_/I_d_, the overdrive voltage V_dsat_ and their relationships were obtained to size the transistors with appropriate width (W) and length (L).

To summarize, the following course of action will take place in the readout circuit under an avalanche breakdown: integration of the photodiode current; saturation of the CTIA; shutting-off of the HV NMOS; reduction in the diode bias and eventually, quenching. The high voltage bias applied on the APD directly appears at the drain of the HV NMOS. However, the HV CMOS technology used in this circuit allows us to exploit the HV NMOS for this purpose. Finally, after the HV NMOS shuts off, the CTIA is reset again which sets the appropriate DC bias conditions for all the transistors in the CTIA to begin the next integration cycle.

### 3.2. Noise Analysis

The CMOS chip described in this paper consists of 8 units of CTIA cell shown in Figure 4. The current design is a lead-in step towards developing mature readouts for large-array GaN APDs in future. Although the design is not noise-optimized, a preliminary analysis is made, identifying several noise sources in the sensor, thus helping set precise specifications for the next-generation readout.

Temporal noise and fixed pattern noise are the major sources of noise in an image sensor [13,14]. For the GaN-CMOS sensor described in this paper, the GaN devices exhibit large performance variation. In the readout circuit, every channel in the 1 × 8 array is read out independently without any particular technique to minimize fixed pattern noise as any spatial variation observed in the readout array is mainly dominated by the differences in the detector performance. Thus, noise analysis in this paper is focused only on the temporal noise from the implemented CMOS readout. The temporal noise sources include the read noise (including amplifier noise), reset noise arising from the reset action on the CTIA feedback and, shot noise. Read noise which also comprises of the CTIA thermal noise is primarily dictated by the input PMOS transistor MP1 in Figure 4, the small-signal model of which is shown below.

**Figure 5:**
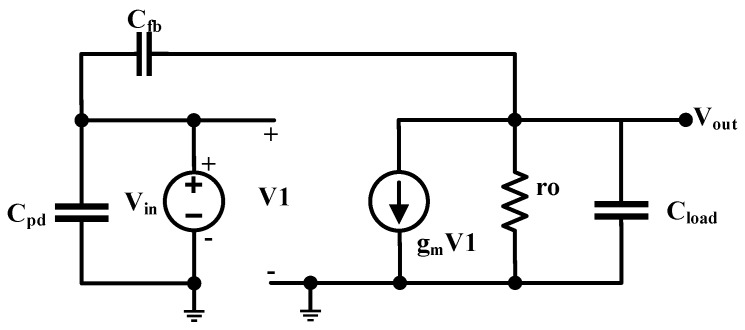
CTIA small-signal model- PMOS input transistor.

The input referred noise voltage per unit bandwidth of this transistor is given as follows [15].

(3) $$\bar{V_{n,in}^{2}}=4kT\frac{\gamma }{g_{m}}$$

where k is the Boltzmann constant, T is the absolute temperature and γ is a constant which is assumed to be 2 for the 0.35 µm process used in this work. From Figure 5, one can deduce the output referred noise density as follows.

(4) $$\bar{V_{n,o}^{2}}=\int_{-\infty }^{+\infty }\bar{V_{n,in}^{2}}|H\left(f\right){|}^{2}df$$

where H(f) = V_out_/V_in_ is the transfer function of the CTIA. In the frequency domain, the small-signal model can be described as,

(5) $$sC_{pd}\left(V1-V_{in}\right)+g_{m}V1+V_{out}\left(\frac{1}{r_{out}}+sC_{load}\right)=0$$

Given that,

(6) $$V1-V_{in}=V_{out}\frac{C_{fb}}{C_{fb}+C_{pd}}$$

and assuming that open loop gain g_m_ro >> C_pd_/C_fb_, Equation (4) can now be reduced to,

(7) $$\bar{V_{n,o}^{2}}=\frac{8kT}{\left(\frac{C_{fb}}{C_{fb}+C_{pd}}\right)\left(C_{load}+\frac{C_{fb}C_{pd}}{C_{fb}+C_{pd}}\right)}$$

Considering one of the possible feedback capacitances (to be able to compare directly with measurement results) and assuming C_fb_ = 400 fF, C_pd_ = 2 pF and C_load_ = 20 pF results in an output referred noise voltage of about 90 µV.

The CTIA also contributes to the reset noise arising from the release of reset switch at the start of every integration cycle. For a given C_fb_, the reset noise voltage is estimated as, $\sqrt{kT/Cfb}$ [16]. Thus, for C_fb_ = 400 fF, $\sqrt{kT/Cfb}\approx$ 100 µV was theoretically estimated.

## 4. Measurement Results

**Figure 6:**
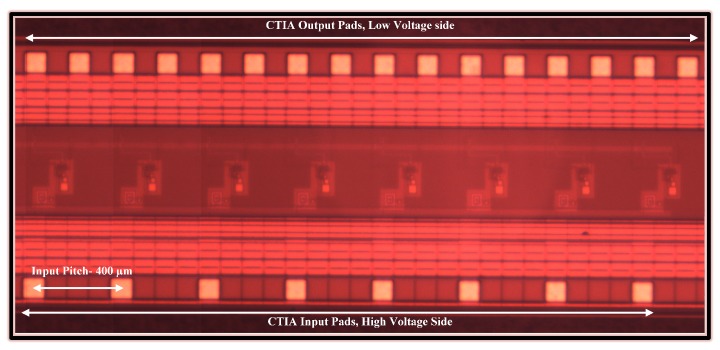
Chip photomicrograph: Eight CTIA unit cells can be identified with their input pads on the bottom side and output pads on the top.

The readout chip is designed and fabricated in 0.35 µm HV CMOS technology. The chip consists of 8 units of the CTIA cell shown in Figure 4 which can also be identified in the photomicrograph of the chip shown below in Figure 6. At a supply voltage of 3.3 V, every unit consumes about 198 µW, thus resulting in a total power consumption of about 1.5 mW for the 1 × 8 CTIA array. As seen in Section 3.1.1, the high voltage which is applied to reverse bias the GaN APD is presented directly at the inputs of the readout circuit if the breakdown mechanism collapses the voltage across the APD. In order to account for such a possibility, the input pads are laid on one side, providing isolation to the low voltage output pads on the other side. The pitch is 400 µm in accordance with the pitch of the detector array.

Two test setups were built in order to characterize the CMOS readout independently and to measure the I–V characteristics of the GaN devices using the CMOS readout chip. In the first setup, a constant current was obtained using a voltage source and a series resistance ≈20 MΩ; the voltage source was swept to perform measurements over varying input current conditions and extract the gain parameters, effective feedback capacitances and slew rate of the CTIA along with the verification of voltage limiting functionality provided by the HV NMOS. In the second test setup, the GaN APDs were connected to the readout chip and a similar measurement was performed.

### 4.1. CTIA Characterization Results

**Figure 7:**
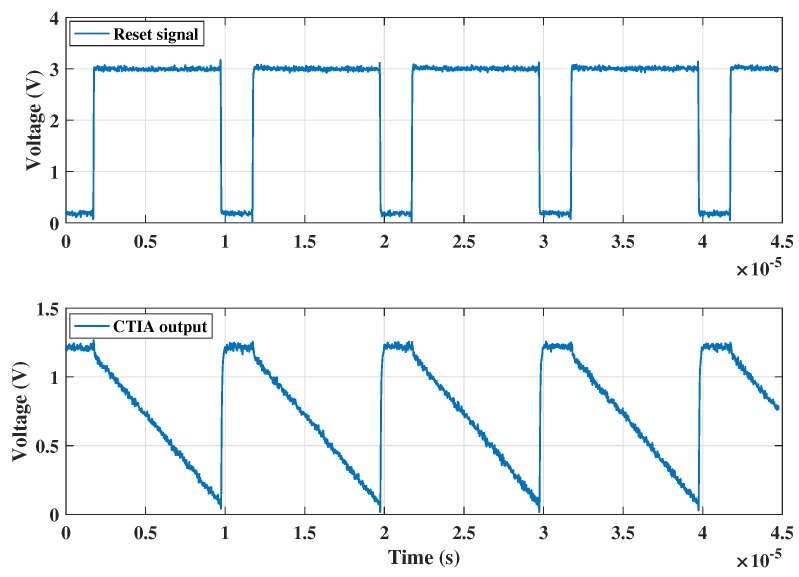
Typical CTIA operation: Reset signal and CTIA output voltage waveforms.

The transient results showing the typical working of the CTIA (as explained in Section 3.1.1) is shown below in Figure 7 over four integration cycles. As can be seen, when the reset voltage at the gate of the PMOS transitor MP_reset_ is 0 V (denoted in Figure 3 and Figure 4), the output voltage V_out_1 is equal to V_b_, which is set according to the DC bias condition applied, 1.2 V. When the reset voltage is pulled up, the feedback path of the CTIA opens and the integration of the incoming current on the feedback capacitor results in a negative ramp (because of a positive current flowing into the CTIA) at the output of the CTIA.

#### 4.1.1. CTIA Transient Behavior

The voltage source, V_s_, connected to a series resistance, R_in_, of approximately 20 MΩ was swept from 2.8–30 V, providing equivalent input currents to the CTIA, equal to $\frac{V_{s}-V_{b}}{R_{in}}$, ranging from 20 nA–0.8 µA. The CTIA output waveform data (at node V_out_1 in Figure 4) was obtained from the oscilloscope for varying input conditions. Differentiating Equation (2) with respect to time results in the following.

(8) $$\frac{dV_{out}}{dt}=\frac{I_{pd}}{C_{fb}}$$

The term $\frac{dV_{out}}{dt}$ basically indicates the slope of the CTIA output voltage waveform captured on the oscilloscope.

**Figure 8:**
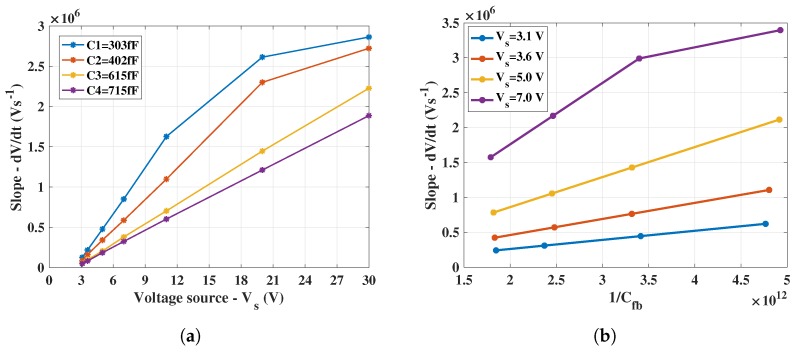
CTIA transient behavior: (**a**) slope versus voltage source, V_s_; and (**b**) slope versus 1/C_fb_.

The slopes were obtained for increasing values of V_s_ (i.e., voltage source connected to the series resistance, R_in_) and thus, equivalently, increasing input currents for the 4 possible feedback capacitor combination; the result of which is shown in Figure 8a. The expected lower slope for higher feedback capacitances (C3 and C4), arising from the higher integration time is also evident, compared to higher slopes for lower feedback capacitances (C1 and C2). According to Equation (8), the obtained slope increases for increasing values of the voltage, V_s_, (and input currents). Similarly, the direct proportionality of the slope with increasing $\frac{1}{C_{fb}}$ is also evident in Figure 8b.

#### 4.1.2. Slew Rate

**Figure 9:**
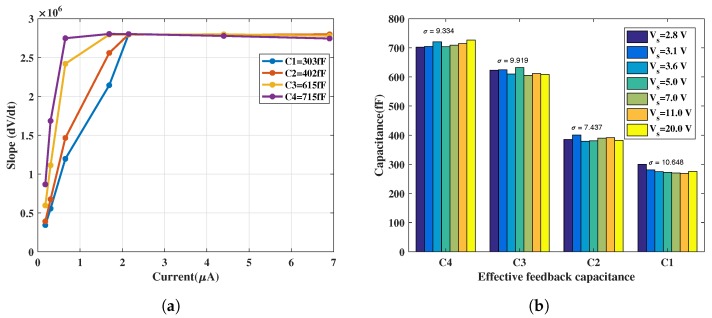
CTIA extracted parameters from transient measurement: (**a**) CTIA slope under higher input currents; and (**b**) variation in measured feedback capacitances (labels as mentioned in Section 3.1.1) .

In Figure 8a, it can be observed that, for lower feedback capacitances (C1 and C2), the slope of the output from the source follower (at node V_out_1 in Figure 4) saturates for higher input currents while the slope is linear for the entire sweep range in case of C3 and C4. Similarly, the inverse trend in the slope saturates for higher input voltage in Figure 8b. Figure 9a shows the measured slope, $\frac{dV_{out}}{dt}$, for input currents up to 7 µA calculated from CTIA output waveform. It was observed that, for input currents approximately above 1 µA, the slopes obtained with all four feedback capacitances saturated to about ≈2.8 V/µs; this value also provided the slew rate of the CTIA. Thus, in Figure 8, the slope saturates as it approaches the slew rate point. This saturation at the output occurred mainly because of the source follower which cannot sink input currents beyond 1.5 µA.

Geiger mode is an important region of operation to assess single-photon sensitivity in photodiodes. In order to assess the suitability of GaN APDs for operation in the Geiger mode, it is necessary to bias them well above voltages >80 V. In this region of operation, the avalanche current increases substantially (to several microamperes) which requires the readout circuit to accommodate higher input currents. Slew rate is thus, an important parameter which indicates the upper limit on the pulldown current of the output source follower in the readout circuit. As seen in Figure 9a, it is evident that the current design limits the input current to about 1.5 µA. Further versions of the readout chip will thus, require improvement to overcome this limitation to examine Geiger mode in these APDs.

#### 4.1.3. Effective Feedback Capacitances

The output slope values extracted through results shown in Figure 8a were utilized along with corresponding input currents to calculate the effective feedback capacitances based on Equation (8). The nominal values of the feedback capacitances from the design (C1 = 50 fF, C2 = 150 fF, C3 = 350 fF, C4 = 450 fF) are lower compared to the values extracted from the gain measurement (shown in Figure 9b); a reason for this deviation between nominal and measured C_fb_ values is the underestimation of parasitics during post-layout simulations. However, the relative differences between the measured feedback capacitances (C2 − C1 = 100 fF, C3 − C2 = 200 fF, C4 − C3 = 100 fF) align with the design specifications described in Section 3.1.1. The bar chart in Figure 9b shows that the measured values result in a standard deviation of about 10.6 fF for the smallest feedback capacitance (≈300 fF), indicating that there is only minimal variation in the extracted values from the measurement.

#### 4.1.4. Charge-to-Voltage Conversion Factor

Charge-to-voltage conversion factor (CVF) (also referred as conversion gain) is a common characterization parameter for CTIA-based circuits. It is basically the ratio between the CTIA output voltage and amount of electrons being transferred over the CTIA feedback capacitance to the output, usually expressed in µV/e^−^. In a CTIA circuit, the feedback capacitor sets this figure, for a constant bias condition on the GaN APD.

(9) $$\Delta V_{out}=\frac{\Delta Q_{in}}{C_{fb}}$$

From the results obtained in Section 4.1.3 for the four feedback capacitances, a CVF of 0.39 µV/e^−^ was obtained at a mean feedback capacitance value of 402 fF.

#### 4.1.5. Voltage Limiter Functionality

**Figure 10:**
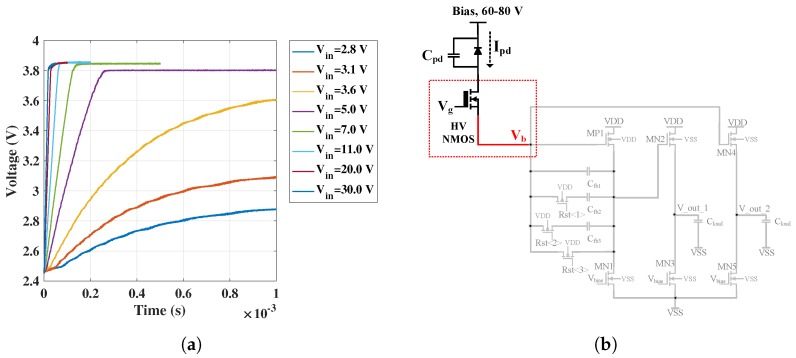
Voltage limiting functionality: (**a**) rise in the CTIA input node V_b_; and (**b**) readout schematic- Input node highlighted.

The HV NMOS introduced at the input of the CTIA acts like a voltage limiter as explained in Section 3.1.1. This functionality was tested in the readout circuit for increasing input currents by monitoring the node V_b_ (as denoted in Figure 4) after the CTIA saturated. Figure 10a shows the input node (V_b_) signal obtained from the oscilloscope. HV NMOS is biased with 4.5 V DC source such that its V_gs_ lies approximately 1 V above its threshold voltage (V_gs_ − V_th_≈ 1 V). After the CTIA saturates upon completion of integration, V_b_ starts rising; this is depicted in Figure 10a. This rise continues until a point when V_gs_ < V_th_ and the HV NMOS ceases to conduct. Thereafter, V_b_ saturates; in this case, the saturation occurs at ≈3.8 V. It will be seen in subsequent sections that this functionality successfully allowed the possibility of diode-bias voltages as high as 80V without damaging the low-voltage CMOS integrated circuit (IC).

### 4.2. GaN + CMOS Measurement Results—Demonstration of UV Sensitivity

**Figure 11:**
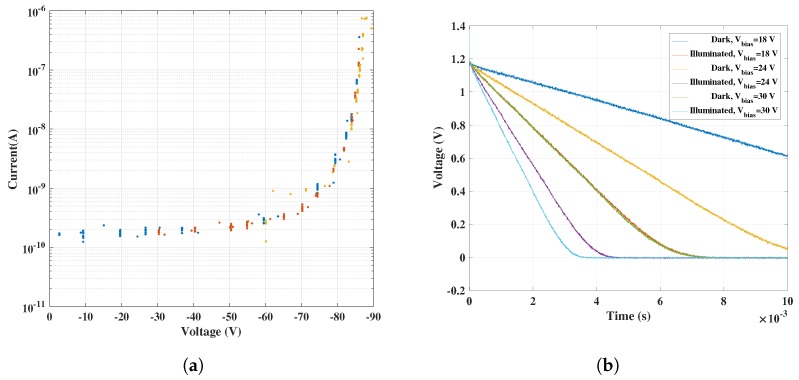
Characteristics of GaN sensor obtained using the CMOS readout circuit: (**a**) extracted I–V curve of a GaN APD; and (**b**) CTIA oscilloscope waveforms.

The standalone characterization of the CTIA circuit was followed by measurements in combination with GaN APDs connected at the input of the CTIA. The CTIA output waveform from the oscilloscope was used along with the feedback capacitance to extract the effective APD currents. The APD reverse bias voltage was swept up to 90 V and the I–V characteristics were obtained as shown in Figure 11a. As can be seen, there is an exponential rise in the current as the APD avalanches beyond 80 V. Additionally, the APD was also illuminated using an available UV LED source to demonstrate the ability of capturing UV sensitivity using the readout circuit. The raw oscilloscope waveforms from CTIA (obtained at node V_out_1 in Figure 11b) are shown in Figure 11b for three different APD bias voltages.

**Figure 12:**
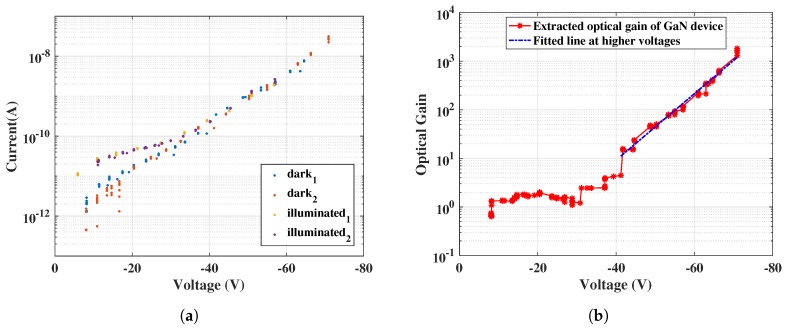
GaN sensor characteristics under UV illumination. (**a**) It can be seen that, for lower voltages, the dark current is lower than the photocurrent by a factor of 10, while, for higher voltages (>40 V), the dark current also increases as the APD starts avalanching. This characteristic is similar to results shown in Figure 1b. (**a**) I–V curve under UV illumination; and (**b**) optical gain estimated from Figure 12a.

Although we are able to distinguish the dark and the photocurrent in the UV region, the results also suggest that the tested GaN APDs have higher dark currents. From the slope values of results in Figure 11b, the equivalent photodiode currents were extracted and the I–V curve was plotted for increasing reverse bias voltages in Figure 12a. Different colors in Figure 11a and Figure 12a indicate results from different data sets with the same input condition. This was done in order to confirm reproducibility of the measurement.

Further, the avalanche gain is also estimated using a method described in [17], as follows:

(10) $$Avalanche\;gain=\frac{I_{pd}-I_{dark}}{I_{pd\_nogain}-I_{dark\_nogain}}$$

where I_pd_ is the photodiode current, I_dark_ is the dark current and I_pd_nogain_ and I_dark_nogain_ are their average values at the unity-gain point. The avalanche gain achieved was about 10 at lower bias voltages, while increasing to 10^3^ at 70 V, shown in Figure 12b. As can be seen, the results obtained so far successfully verify the voltage limiting functionality of the HV-NMOS, by allowing reverse bias voltages up to ≈80 V.

### 4.3. Noise Measurement

The temporal noise sources identified in Section 3.2 are measured and the obtained results are discussed in this section.

#### Conversion Gain and Read Noise

The APD, when biased at certain voltage, contributes to the shot noise in the sensor and the readout chip contributes to the read noise of the sensor. Shot noise is basically the temporal variation in the electron-hole pairs generated inside the APD due to random arrival of the impinging photons which increases in proportion to the incident photon level [18]. The arrival of photons is governed by Poisson statistics such that the corresponding variance in number of photons, *n*, is given by $\sigma_{n}^{2}=\bar{n}$. For the sensor with a gain, G (which includes the CTIA conversion gain and the APD avalanche gain), the output noise voltage $V_{out}$ approaches

(11) $$V_{out}=G.n$$

The shot noise variance, $\sigma_{sn}^{2}$, at the output is then equal to the mean number of incident photons, $\bar{n}$, multiplied by gain, G. Furthermore, the readout array contributes to the read noise (including the amplifier noise). Thus, the final noise variance, $\sigma_{n}^{2}$, can then be represented as,

(12) $$\sigma_{n}^{2}=\sigma_{o}^{2}+G.\bar{V_{out}}=\sigma_{o}^{2}+G.\bar{n}$$

where the intercept of Equation (12), $\sigma_{o}^{2}$, gives the read noise power [19]. The plot of variance versus mean output voltage (commonly also referred to as the photon-transfer curve (PTC)) is thus, very useful to extract conversion gain and noise contributions of the readout. Further, there is also noise due to the random fluctuations in the gain, G. This produces a gain dependent multiplicative “Excess Noise Factor” which introduces a scale factor to the PTC curve [20]; however, this is assumed to be small for low gains where we are operating. The PTC plot is obtained by measuring the CTIA with an APD biased at 40 V under typical indoor illumination. Gain and noise parameters are extracted and the results obtained are compared with those obtained in Section 4.1.4.

**Figure 13:**
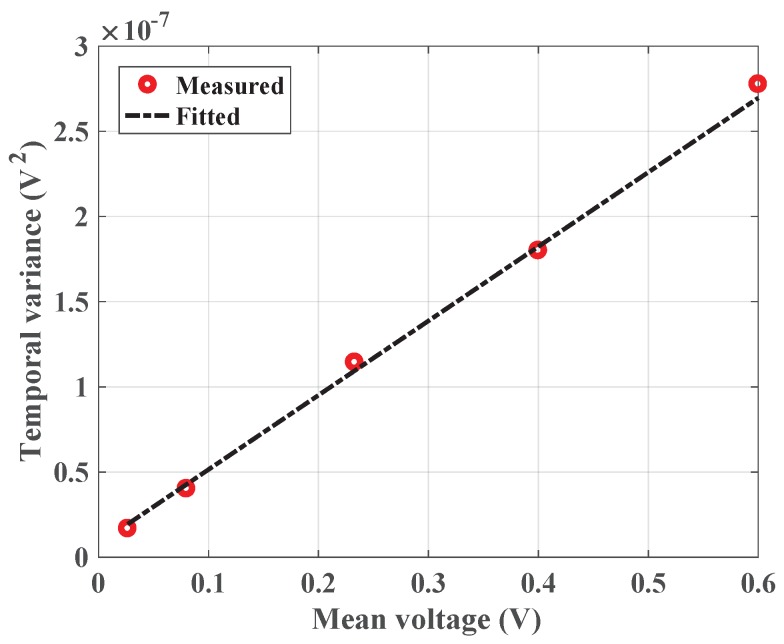
Temporal variance versus mean output voltage extracted from measurement.

The variance and mean were extracted for the difference obtained between the first sample (S1) and every subsequent sample (S1−S2, S1−S3 etc.) in the measured data. Interquartile estimate of variance was used to reduce the effects of outliers on the sampled data. The plot in Figure 13 shows the measured temporal variance for increasing mean output voltages against a fitted line.

The slope of the plot in Figure 13 gives the value of conversion gain. For a feedback capacitance of 402 fF used in this measurement, the obtained conversion gain is 0.43 µV/e^−^ from the slope. This value, as expected, is close to what is estimated in Section 4.1.4 from the measured feedback capacitance of 402 fF (0.39 µV/e^−^), with <10% deviation, thus, confirming the accuracy of the measurement. The intercept obtained from the line plot results in a read noise voltage of 88 µV. It must be noted that while the read noise includes thermal noise and 1/f noise, the latter contributes little to the total noise at the output. This is because the measurements are made by subtracting the first sample (S1) from every subsequent sample as mentioned above; this method is a form of correlated double sampling (CDS) performed off-chip which is a standard approach to combating 1/f noise.

#### CTIA- Reset Noise

The variation in the voltage level after the release of the reset switch determines the reset noise of the CTIA. The data set used to plot Figure 13 is used to also estimate this value. The variance at the CTIA output is a combination of the correlated noise from the reset mechanism and the uncorrelated read noise. The read noise obtained from the mean-variance plot is subtracted from the variance of sample, S1, acquired right after the release of reset switch [13]. The read noise estimated in the previous section is however, measured from a pair of samples. Thus, to estimate the reset noise, only half of the read noise is considered. This resulted in a reset noise voltage of 121.6 µV. Comparing this with the theoretical value (also as calculated in Section 3.2— $\sqrt{kT/Cfb}\;\approx$ 101.2 µV and C_fb_ = 402 fF, used in this measurement) shows that the noise floor is dominated by the ADC quantization noise (≈112 µV) from the oscilloscope. Since there was no ADC in the readout chip, the quantization noise from the oscilloscope limited the measurable noise voltage; for the same reason, the thermal noise estimated for the CTIA in Section 3.2 could not be measured directly and only the read noise from the mean-variance method was estimated, seen in Figure 13.

## 5. Next-Generation Readout Improvements

Table 1 summarizes the measurement results obtained so far.

The CTIA implemented in this readout chip is a proof-of-concept with GaN APDs. Although the basic functionality of the readout along with GaN APDs was successfully verified with measurements, there are several improvements which can be made in the next version of the readout. The operational bandwidth of the CTIA needs to be improved; the bias current can thus be increased; including a cascode transistor is also an option, which will lower down the capacitance at the drain of the PMOS input transistor. However, the trade-off between increased output impedance and lower cutoff frequency needs more analysis at design level. Current version of the chip cannot draw currents above 1.5 µA, as seen in Section 4.1.2. To accommodate higher currents, primarily required also to operate GaN APD at higher voltages and monitor avalanches, it is necessary to improve the current sinking capabilities of the source follower stage. Furthermore, a programmable reset needs to be implemented in the next chip, given a clearer understanding of the timing behavior from the transient measurement results. An integrated analog-to-digital converter (ADC) is one of the future additions to provide on-chip solution for sampling the CTIA output voltage. A target SNR of 50 dB for the next chip results in an achievable loop gain of about 54 dB, making it suitable for this implementation.

## 6. Hybridizing with 3D Stacked Technology—A Perspective

**Figure 14:**
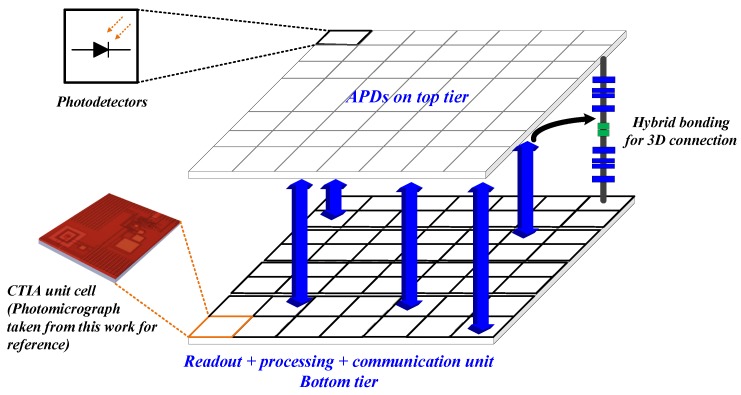
Conceptual representation of 3D stacking.

There has been a growing interest in 3D integration technology in the last few years, thanks to the wafer-level stacking possible with this technology [8,9,10,11]. Figure 14 visualizes the 3D stacking concept with photodetectors laid on the top tier and readout, processing and communication unit on the bottom tier.

A direct vertical tier-tier connection not only reduces the interconnection parasitics compared to 2D wire-bonded connection but also permits massive parallelization between the two tiers. The work done so far is a proof-of-concept demonstrating the feasibility of our approach of combining GaN APDs with CMOS circuits. The results from this work can lead towards a 3D-integrated chip in future when the APDs can be stacked on top of the custom CMOS circuit. Furthermore, based on the compatibility of the detector substrate with silicon, the hybrid concept can allow easy porting of the CMOS circuits to any kind of photodetector, including the GaN-based detectors discussed here.

## 7. Conclusions

We reported on a 1 × 8 linear array of capacitive transimpedance amplifiers implemented in CMOS technology, as readouts for GaN APDs. We successfully tested the readout circuits with GaN APDs and provided a viable solution to operate the APDs at very high reverse bias voltages (≈80 V) without damaging the low-voltage CMOS circuit. The results from this work will, in the future, enable easy characterization of the APDs with the current readout, while a next-generation of the readout is also being planned, which will enable higher avalanche gains. Future work will also include performing measurements in radiation-environment to ensure functionality in space applications.

## Figures and Tables

**Table 1:** Performance summary.

Parameter	Results
Photodetector technology	GaN avalanche photodiode
APD bias voltage	0–80 V, proportional-mode
Readout technology	0.35 µm HV CMOS, Supply voltage = 3.3 V
Readout topology	Capacitive transimpedance amplifier (CTIA)
CTIA array size	1 × 8
CTIA area	≈ 5 mm × 1 mm
Input current range	150 pA–1.5 µA
Slew rate	2.8 V/µs
Conversion gain	0.43 µV/e^−^
CTIA read noise	88 µV
CTIA reset noise	121 µV
Power consumption	1.5 mW
Avalanche Gain	$10^{3}$

## Abbreviations

UV	Ultraviolet
APDs	Avalanche photodiodes
GaN	Gallium nitride
CMOS	Complementary metal-oxide-semiconductor
SNR	Signal-to-noise ratio
TIA	Transimpedance amplifier
RTIA	Resistive transimpedance amplifier
CTIA	Capacitive transimpedance amplifier
HV	High voltage
CVF	Charge-to-voltage conversion factor
ADC	Analog-to-digital converter
CDS	correlated double sampling
IC	Integrated circuit
